# Homozygous *TBC1D24* Mutation in a Case of Epilepsia Partialis Continua

**DOI:** 10.3389/fneur.2017.00750

**Published:** 2018-01-24

**Authors:** Qilin Zhou, Yicong Lin, Jing Ye, Liping Li, Ningning Hu, Di Wang, Yuping Wang

**Affiliations:** ^1^Department of Neurology, Xuanwu Hospital, Capital Medical University, Beijing, China; ^2^The Beijing Key Laboratory of Neuromodulation, Beijing, China; ^3^Center of Epilepsy, Beijing Institute for Brain Disorders, Capital Medical University, Beijing, China

**Keywords:** *TBC1D24* gene, epilepsia partialis continua, homozygous mutation, morphometric analysis program, MRI post-processing technique

## Abstract

*TBC1D24* mutation-related epileptic syndrome includes a wide spectrum of epilepsies. We describe a case with a homozygous *TBC1D24* mutation inherited from consanguineous parents. The patient manifested epilepsia partialis continua (EPC) and rare secondary generalized tonic–clonic seizure without intellectual disability or developmental delay. EPC, which involved focal limbs, came with waking and went with sleep. The genetic analysis reported a novel mutation in the *TBC1D24* gene, c.229_240del (p.82_84del). The homozygous mutation was inherited from her healthy parents who were heterozygous. Morphometric analysis program (MAP), an MRI post-processing technique, was used and detected a subtle abnormality of the brain. A comprehensive analysis based on semiology, electroencephalogram, somatosensory-evoked potential, and MAP suggested a potential focal structural abnormality. This case indicates a possible correlation between the *TBC1D24* mutation and brain development abnormality.

## Introduction

*TBC1D24* mutation-related epileptic syndrome has various clinical characteristics. The correlation between phenotype and genotype is unclear. We reported a case of the homozygous *TBC1D24* mutation, which was inherited from heterozygous parents. The patient’s distinct manifestation was epilepsia partialis continua (EPC). We also used the morphometric analysis program (MAP), an MRI post-processing technique, to detect a subtle lesion of the brain, aiming to describe the syndrome by genotype, phenotype, neuroimaging, and electrophysiology aspects.

## Case Report

A 16-year-old female from a Chinese family with consanguineous parents, developed her first episode of jerks after a cold 11 years ago. The jerks involved right foot and leg without preceding sensation and was not relieved until she was given diazepam intravenously. The antiepileptic drugs (AEDs) were provided, but the episodes still occurred 3–4 times/year. The jerks were usually triggered by fatigue, emotions, or fever and lasted for minutes to hours. It could only be controlled by intravenous diazepam. She was diagnosed with “psychogenic attack” 6 years ago and was weaned off AEDs. The “psychogenic attacks” continued. Three years ago, the patient experienced loss of consciousness for minutes after right arm jerks. Six days before the admission, she developed right foot jerks after a long walk, but the jerks could not be relieved completely by diazepam. The jerks stopped with sleep without any treatment and reoccurred with waking. During the course of the disease, the patient had no intellectual disability or developmental delay, hearing loss, onychodystrophy, osteodystrophy, paresthesia, or cerebellar ataxia. She felt fatigue after running, long-distance walking, and climbing stairs. She had no remarkable medical history. Another individual in her family had similar paroxysmal episodes of leg jerks. Upon physical examination, no neurological deficit was found. MMSE test scored 29. The audiometry test was normal. The serum autoimmune panel, including anti-streptolysin “O,” C-reactive protein, rheumatoid factors, immunoglobulin G, immunoglobulin M, immunoglobulin A, alexin C3, alexin C4, erythrocyte sedimentation rate, anticardiolipin antibody, anti-nuclear antibody, anti-neutrophil cytoplasmic antibody, thyroid peroxidase antibody, and thyroglobulin antibody, was negative. Both amino acid and organic acid metabolism tests of serum and urine were negative.

**Figure 1:**
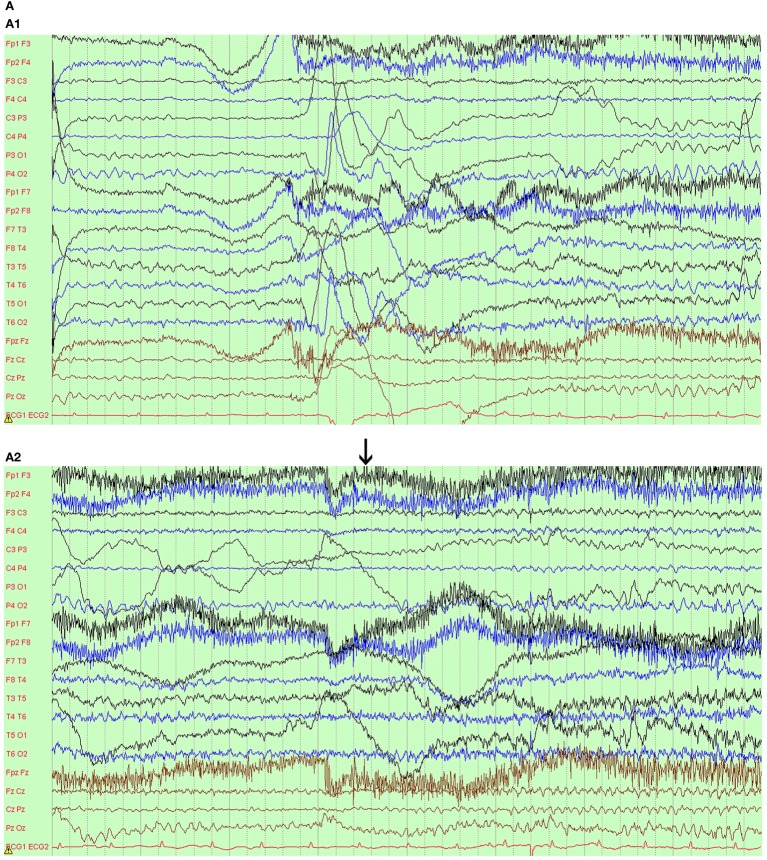

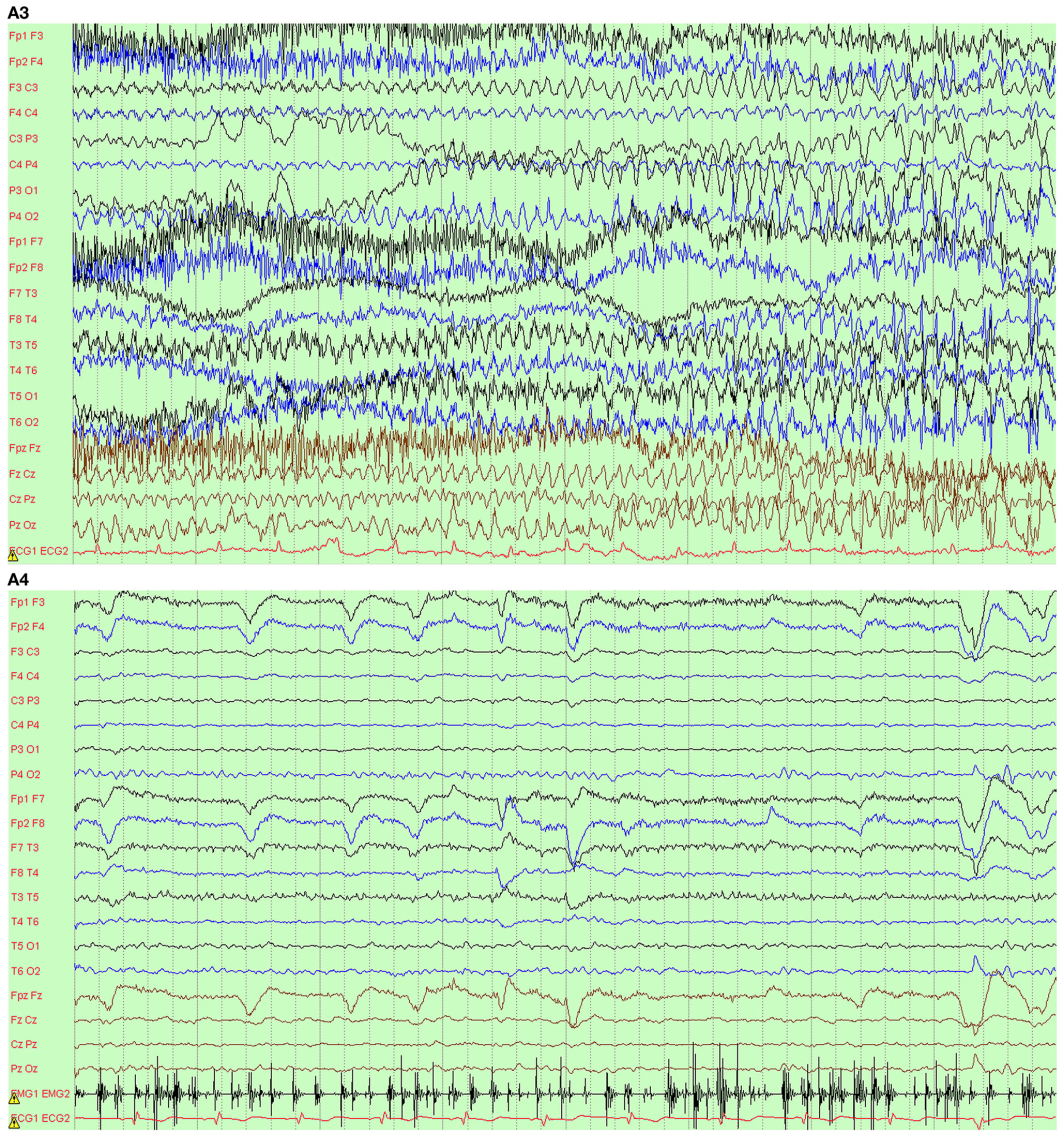

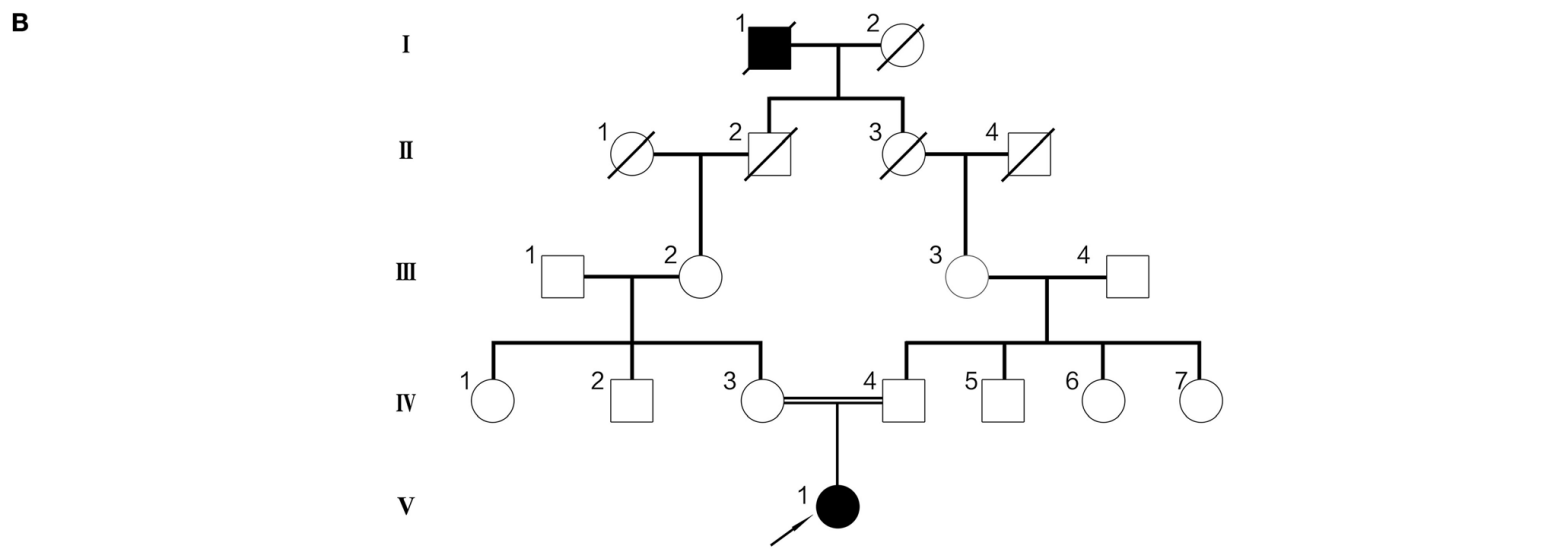
Ictal electroencephalogram (EEG) and pedigree of the family. **(A)** Ictal EEG with left frontocentral origination (arrow). A1–A3 show EEG evolution. A4 shows epilepsia partialis continua of the right lower limb without visible EEG change. **(B)** Pedigree of the family. The black square/circle indicates patients with the epilepsy phenotype; the double horizontal lines denote consanguineous marriage; the arrow shows the proband.

Continuous video electroencephalogram (EEG) monitoring showed normal background without EEG change during leg jerking. One event was captured. The patient’s head and eyes deviated to the right with the right arm rising and the leg stretching straight. Then, the convulsion involved all limbs and lasted 20 s. During the event, she was alert but could not speak. The EEG showed rhythmic activity from the left frontocentral and midline regions. The activity quickly spread to the contralateral side with increased amplitude (Figure 1A). The brain MRI was reported normal. The somatosensory-evoked potential (SEP) showed remarkable reduced amplitude in the left cortex when right leg was tested. The upper limbs’ SEPs were normal. Genetic analysis through an epilepsy panel of 480 genes by Kangso Medical Inspection reported a homozygous mutation in the *TBC1D24* gene, c.229_240del (p.82_84del). The homozygous mutation was inherited from her healthy parents who were heterozygous, which was consistent with autosomal recessive inheritance (Figure 1B and Supplementary Figure S1). We could not predict protein function related to this mutation through SIFT, Polyphen2, or Mutation Taster analysis software.

**Figure 2:**
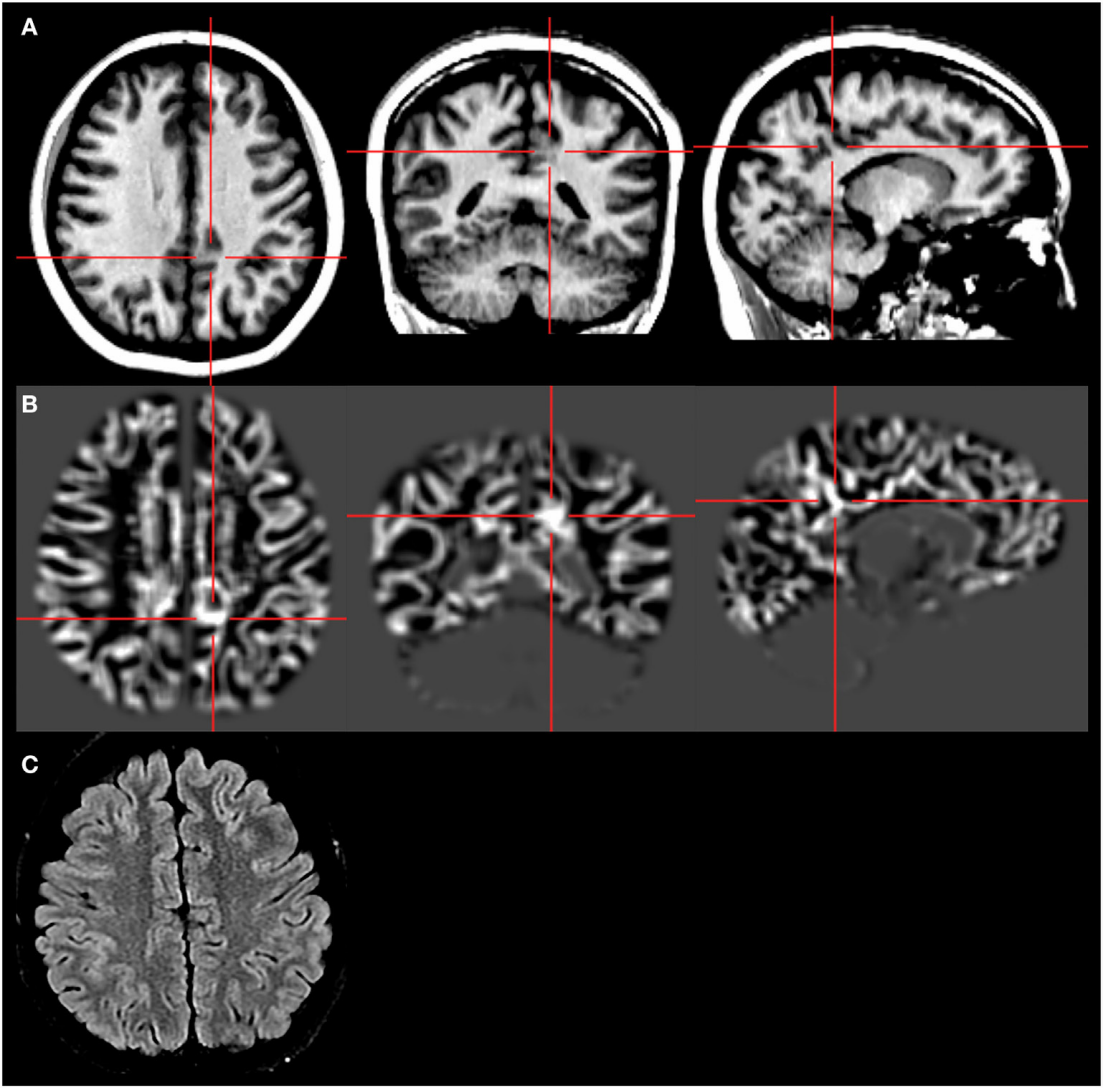
Morphometric analysis program (MAP) result. The crosshairs indicate the location of the MAP^+^ candidate. *First row* **(A)**: T1-weighted magnetization prepared rapid acquisition with gradient echo images. *Second row* **(B)**: gray–white matter junction *z*-score file, as the output of MAP processing of the T1-weighted image shown in the first row. *Third row* **(C)**: T2-weighted fluid-attenuated inversion recovery images, chosen to best depict the MAP^+^ candidate. This was the best available image due to sub-optimal slice selection. There are no corresponding T2 changes.

Morphometric analysis program was performed on 3-T T1-weighted images using SPM12 toolbox in MATLAB R2011b following previously established methods (1). The computed output was a junction map, which highlighted abnormal structures deviating from the average normal structures based on *z*-score. The normal database was provided along with the MAP program. The highlighted region might correlate with gray–white blurring on the underlying MRI. The MAP analysis identified a subtle abnormality candidate with a *z*-score over 4, a subtle gray–white blurring in the left medial parietal region (Figure 2).

The patient was given levetiracetam with a gradually increasing dosage to 1.25 g twice/day and sodium valproate with a dosage of 0.5 g twice/day and was completely relieved several days later. Upon follow-up, the patient was weaned off the sodium valproate and did not report any symptoms in the 10-month follow-up.

Both patient and parents provided written informed consent for the study and publication.

## Discussion

We report here a novel homozygous mutation in *TBC1D24*: the c.229_240del (p.82_84del). The variants were inherited from the patient’s consanguineous parents who were healthy. Another family member, who probably carried the homozygous gene mutation, had similar symptoms. The phenotypes were characterized as EPC and rare secondary tonic–clonic seizure, without intellectual disability or hearing loss. EPC resolved with sleep and reappeared with waking and was not associated with EEG changes. The tonic–clonic seizure probably originated from the left central region. The clinical manifestation was consistent with reported cases of the *TBC1D24* mutation. Balestrini et al. (2) summarized the clinical phenotypes and genotypes of 48 patients with *TBC1D24* mutation-related epileptic syndrome. The authors found that this syndrome comprised a group of epilepsies with clinical heterogeneity. Most patients suffered the first seizure between the ages of 7 months and 8 years. The most common seizure types were myoclonus or clonic seizures. Myoclonus could be focal or generalized, always appeared in clusters and sustained for many days. In 2017, two articles expanded the phenotypic spectrum. Ragona et al. (3) reported a *TBC1D24* mutation case that was related to alternating hemiplegia. Banuelos et al. (4) described a *TBC1D24* mutation that was linked to tonic–clonic and myoclonic epilepsy in autosomal dominant inheritance, psychosis, parkinsonism, and intelligent obstacle with autosomal recessive inheritance. Trivisano et al. (5) reported two *TBC1D24* mutation patients died from sudden unexpected death (SUDEP). The mechanism of SUDEP is still unclear. Some risk factors such as generalized tonic–clonic seizures, nocturnal seizures, early onset epilepsies, and polytherapy may cause SUDEP in patients (5, 6).

The *TBC1D24* gene encodes a protein with an N-terminal Tre2/Bub2/Cdc16(TBC) domain, which interacts with GTPases. *TBC1D24* could affect the migration and maturation of neurons by regulating the activity of ADP-ribosylation factor (ARF-6) (7). ARF-6 is a type of GTPase associated with vesicle membrane transport that plays a pivotal role in brain development (8). Therefore, the *TBC1D24* mutation may potentially affect brain development, although the specific mechanism has not yet been clarified. Recently, Fischer et al. (9) found an unanticipated cationic pocket that bound phosphoinositides in a study of the crystal structure of the TBC domain of the *Drosophila* ortholog *Skywalker*, which had the same domain organization as humans. The most common mutations affect the phosphoinositide-binding pocket and inhibit lipid binding, consequently causing impaired synaptic-vesicle trafficking, seizures, and many other neurological defects in flies *via* restricting Skywalker diffusion in presynaptic terminals. The *TBC1D24* gene encoded protein also contains a TLDc domain, which participates in oxidative stress (OS) resistance. The OS response is essential for the dynamic balance of reactive oxygen species (ROS), the important intracellular messengers. The imbalance in the numbers of ROS can do harm to DNA, proteins, and lipids and results in neuronal hyperactivity, synaptic dysfunction, and neuronal loss (10).

In Balestrini’s review (2), 16 out of 48 patients with the *TBC1D24* mutation had visible structural abnormalities on MRI, including delayed myelination, hippocampal sclerosis, vermian hypoplasia, cerebellar atrophy, and cerebellar signal hyperintensity. In our case, the MRI was reported normal. We further performed an MRI post-processing procedure and found a subtle gray–white blurring in the left medial parietal region. The focal feature, based on MAP, SEP, EEG, and semiology, most likely indicated a restricted brain structural abnormality in the left medial centro-parietal region. We hypothesize that the *TBC1D24* mutation might lead to a structural abnormality during brain development. Re-reviewing the MRI, MRI post-processing, or a combination of multi-modality neuroimaging may help detect abnormalities in patients with the *TBC1D24* mutation and probably has implications for elucidating the mechanism of epileptogenesis.

## Conclusion

Our report reveals a novel mutation and expands the genotypic spectrum of *TBC1D24* mutation-related epileptic syndrome. A potential regional epileptogenic brain structural abnormality may develop in cases of *TBC1D24* mutation.

## Ethics Statement

All clinical data in this case report were either provided by the patient and her parents or collected by our team’s members with the consent of them. There was no additional invasive test or experimental drugs used out of order for the patient. A written informed consent was obtained from the patient and her parents for the participation in the study and the publication of this report. The case report is exempt from institutional review board approval.

## Author Contributions

QZ acquired the clinical data, reviewed the literature, and drafted the manuscript. YL designed the study, oversaw data acquisition, supervised the initial drafting, performed post-MRI processing, and critically revised the manuscript. JY and YW analyzed the clinical data and critically revised the manuscript. LL and NH analyzed and interpreted the electrophysiological data and critically revised the manuscript. DW reviewed the literature and revised the manuscript.

## Conflict of Interest Statement

The authors declare that the research was conducted in the absence of any commercial or financial relationships that could be construed as a potential conflict of interest.

## References

[1] Huppertz HJ Grimm C Fauser S Kassubek J Mader I Hochmuth A Enhanced visualization of blurred gray-white matter junctions in focal cortical dysplasia by voxel-based 3D MRI analysis. Epilepsy Res (2005) 67(1–2):35–50. 10.1016/j.eplepsyres.2005.07.009 16171974

[2] Balestrini S Milh M Castiglioni C Luthy K Finelli MJ Verstreken P TBC1D24 genotype-phenotype correlation: epilepsies and other neurologic features. Neurology (2016) 87(1):77–85. 10.1212/WNL.0000000000002807 27281533 PMC4932231

[3] Ragona F Castellotti B Salis B Magri S DiFrancesco JC Nardocci N Alternating hemiplegia and epilepsia partialis continua: a new phenotype for a novel compound TBC1D24 mutation. Seizure (2017) 47:71–3. 10.1016/j.seizure.2017.03.003 28292732

[4] Banuelos E Ramsey K Belnap N Krishnan M Balak C Szelinger S Case report: novel mutations in TBC1D24 are associated with autosomal dominant tonic-clonic and myoclonic epilepsy and recessive parkinsonism, psychosis, and intellectual disability. F1000Res (2017) 6:553. 10.12688/f1000research.10588.1 28663785 PMC5473401

[5] Trivisano M Bellusci M Terracciano A De Palma L Pietrafusa N Valeriani M TBC1D24 gene mutations are associated with high risk of sudden unexpected death. Epilepsy Behav (2017) 72(2017):208–9. 10.1016/j.yebeh.2017.04.010 28606688

[6] Devinsky O Hesdorffer DC Thurman DJ Lhatoo S Richerson G Sudden unexpected death in epilepsy: epidemiology, mechanisms, and prevention. Lancet Neurol (2016) 15:1075–88. 10.1016/S1474-4422(16)30158-2 27571159

[7] Falace A Filipello F La Padula V Vanni N Madia F De Pietri Tonelli D TBC1D24, an ARF6-interacting protein, is mutated in familial infantile myoclonic epilepsy. Am J Hum Genet (2010) 87(3):365–70. 10.1016/j.ajhg.2010.07.020 20727515 PMC2933335

[8] Stenmark H. Rab GTPases as coordinators of vesicle traffic. Nat Rev Mol Cell Bio (2009) 10(8):513–25. 10.1038/nrm2728 19603039

[9] Fischer B Lüthy K Paesmans J Koninck CD Maes I Swerts J Skywalker-TBC1D24 has a lipid-binding pocket mutated in epilepsy and required for synaptic function. Nat Struct Mol Biol (2016) 23(11):965–73. 10.1038/nsmb.3297 27669036

[10] Finelli MJ Oliver PL. TLDc proteins: new players in the oxidative stress response and neurological disease. Mamm Genome (2017) 28:395–406. 10.1007/s00335-017-9706-7 28707022 PMC5614904

