# Supplementary material for: Homozygous TBC1D24 Mutation in a Case of Epilepsia Partialis Continua
Source: Front Neurol. 2018 Jan 24;8:750. doi: 10.3389/fneur.2017.00750 (PMC5787533; doi:10.3389/fneur.2017.00750)
Supplement: Supplementary file 1 [file data_sheet_1.docx]

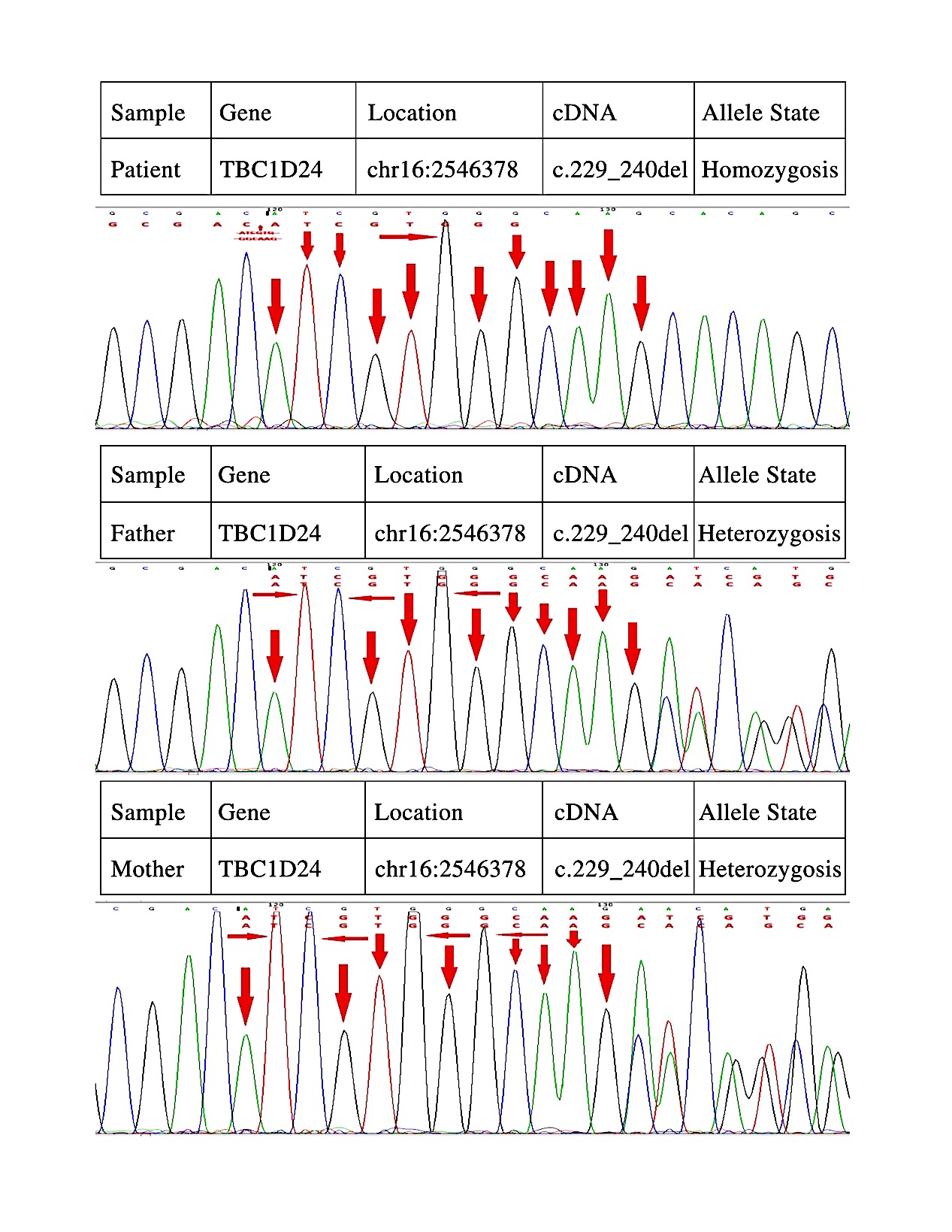


Supplementary Figure 1. In this patient, homozygosis variation c.229_240del(p.81_84del) was identified within TBC1D24 gene, which located in the chr19:13345826. Samples of patient's parents were both heterozygous at the same site.
